# Functional Outcomes of Spherical Pyrocarbon HAPY Metacarpophalangeal Interposition Arthroplasty for Long Fingers: A Retrospective Study of 16 Cases

**DOI:** 10.1016/j.jhsg.2025.100804

**Published:** 2025-08-08

**Authors:** Cerise Gosselin, Hugo Barret, Clémentine Rieussec, Romain Mari, Alexandra Forli, Denis Corcella

**Affiliations:** ∗ Chirurgie Orthopédique et Traumatologique, Centre Hospitalier Universitaire de Purpan, Purpan, Toulouse, France; † Chirurgie Plastique et Réparatrice des Membres – Chirurgie de la Main, Centre Hospitalier Universitaire de Grenoble, La Tronche, France

**Keywords:** Arthroplasty, HAPY implant, Metacarpophalangeal joint, Osteoarthritis, Pyrocarbon

## Abstract

**Purpose:**

Metacarpophalangeal (MCP) joint osteoarthritis is a major cause of pain and functional limitation. When conservative treatment fails, arthroplasty is a surgical option, with silicone implants historically being the most widely used. The HAPY spherical metacarpal pyrocarbon implant is a new prosthetic solution. This study aims to evaluate the clinical and radiological outcomes of this spherical pyrocarbon MCP implant.

**Methods:**

This single-center retrospective study included 16 patients who underwent MCP arthroplasty with the HAPY implant for osteoarthritis with a follow-up of more than 2 years. Clinical evaluation included pain (visual analog scale), range of motion, and disabilities of the arm, shoulder, and hand score, and grip strength. Complications were also recorded. Radiological assessment focused on implant positioning, bone erosion, peri-implant bone remodeling, proximal migration, and material integrity.

**Results:**

After a mean follow-up of 48 months (range: 24–95 months), functional outcomes demonstrated considerable improvement in MCP flexion (41° ± 10° to 64° ± 20°), pain reduction (visual analog scale: 7.4 ± 1 to 0.8 ± 0.9), and improvement in the disabilities of the arm, shoulder, and hand score (42.5 ± 11.3 to 20.2 ± 22.5). Grip strength remained stable. No complications such as dislocation, instability, or infection were reported. Radiological outcomes showed proper implant positioning in 62.5% of cases, with moderate peri-implant bone remodeling and a low incidence of proximal migration or considerable erosion.

**Conclusions:**

The HAPY spherical pyrocarbon interposition arthroplasty for the metacarpophalangeal joint demonstrates satisfactory short- to mid-term outcomes, with consderable improvements in pain relief and joint mobility. Radiological findings support good bone tolerance with signs of progressive integration, despite moderate erosion and bone remodeling in some cases. These results are consistent with outcomes reported for other pyrocarbon implants. A multicenter study with a longer follow-up is needed to confirm the durability and safety of this technique.

**Type of study/level of evidence:**

Therapeutic IV.

Osteoarthritis of the metacarpophalangeal (MCP) joints in long fingers is a major cause of pain and functional loss. When conservative treatment fails, several surgical approaches can be considered, including arthroplasty with silicone, metal polyethylene, or pyrocarbon implants, as well as interposition arthroplasty or arthrodesis in cases of prosthetic failure.^1^, ^2^, ^3^, ^4^, ^5^

Historically, silicone implants (eg, Swanson-type) have been widely used because of their simplicity and biomechanical tolerance.^6^, ^7^, ^8^, ^9^, ^10^, ^11^ However, they exhibit high fracture rates (42% at 10 years and 63% at 17 years), often leading to prosthetic migration and compromised implant survival.^12^ Pyrocarbon implants have emerged as a biomechanically superior alternative, with an elasticity modulus similar to cortical bone, reducing mechanical stress and bone resorption risk.^1^^,^^2^^,^^13^

The HAPY pyrocarbon implant (Tornier-Wright) was developed to offer a better biomechanical solution. This MCP spherical interposition arthroplasty preserves bone stock while providing favorable mechanical properties. Studies on pyrocarbon stemmed MCP arthroplasties have demonstrated considerable pain reduction (visual analog scale [VAS] decreased by 68%), improved MCP flexion (+23°), and high patient satisfaction (>90%) at the mid-term follow-up.^14^^,^^15^ However, some studies have reported asymptomatic bone erosion (3–5 mm) or rare implant fractures, necessitating careful radiographic monitoring.^5^^,^^15^

The objective of this study was to evaluate the clinical and radiological outcomes of MCP arthroplasties using the HAPY spherical pyrocarbon implant.

## Materials and Methods

### Study design

This retrospective single-center study included 16 patients who underwent MCP arthroplasty for osteoarthritis using the HAPY pyrocarbon implant (Tornier-Wright). The inclusion criteria were (1) patients over 18 years old, (2) with severe MCP joint osteoarthritis, (characterized by considerable joint destruction, reduced joint space, and/or osteophyte presence), (3) refractory to conservative treatment (analgesics, injections, and orthotics), and (4) having a minimum clinical and radiographic follow-up of 2 years. The exclusion criteria were patients with rheumatoid arthritis, revision surgeries, or prior surgical interventions on the MCP joint.

### Surgical technique

**Figure 1:**
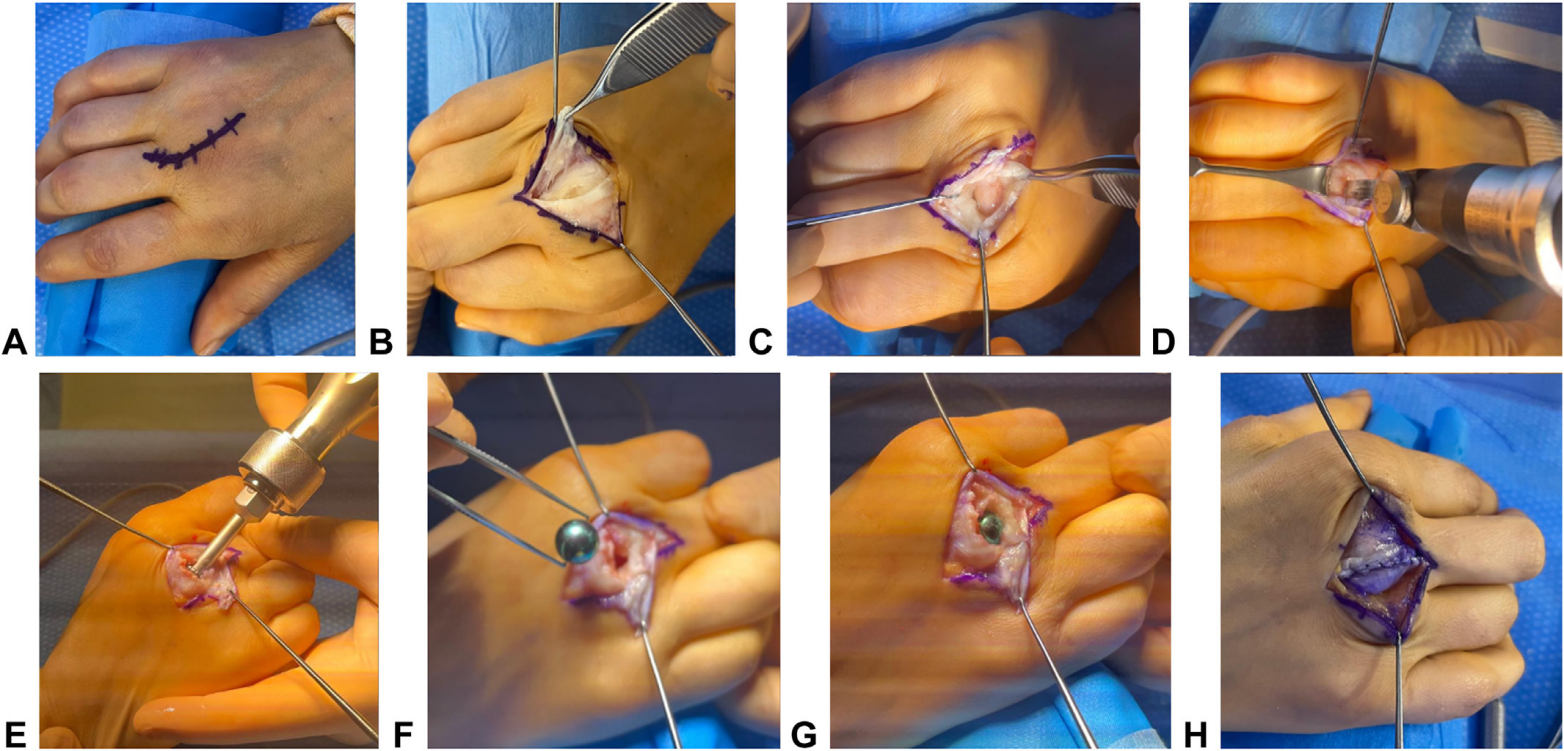
Surgical technique. Curvilinear incision over the MCP joint **A**. Longitudinal incision on the ulnar side of the extensor hood **B**. Transverse incision of the joint capsule **C**. Resection of the metacarpal articular surface using an oscillating saw **D**. Preparation of an intramedullary cavity in the metacarpal using a dedicated burr Photograph of the actual prosthesis **F**. Placement of the trial implant **G**. Closure of the extensor hood with a continuous 3/0 polydioxanone suture **H**.

Metacarpophalangeal arthroplasty using the HAPY pyrocarbon implant is performed under regional anesthesia, with the patient in the supine position and a pneumatic tourniquet applied. A curvilinear incision is made over the affected MCP joint (Fig. 1A). A longitudinal incision (Fig. 1B) is performed on the ulnar side of the extensor hood, preserving 1–2 mm of the extensor tendon to facilitate subsequent suturing. The joint is further exposed by making a transverse incision in the joint capsule (Fig. 1C), ensuring optimal visualization of the MCP articulation.

A resection of the metacarpal articular surface is performed using an oscillating saw (Fig. 1D), with a cut flush to the metacarpal head, just before the insertion of the collateral ligaments, which must be preserved. The anterior osteophyte is resected with a 20° to 30° angled cut to optimize joint space and prevent mechanical interference with the implant. A meticulous bone preparation is carried out using a dedicated burr to create a precise intramedullary cavity in the metacarpal (Fig. 1E). A progressive compactor is then used to condense the cancellous bone and optimize the implant’s primary fixation.

**Figure 2:**
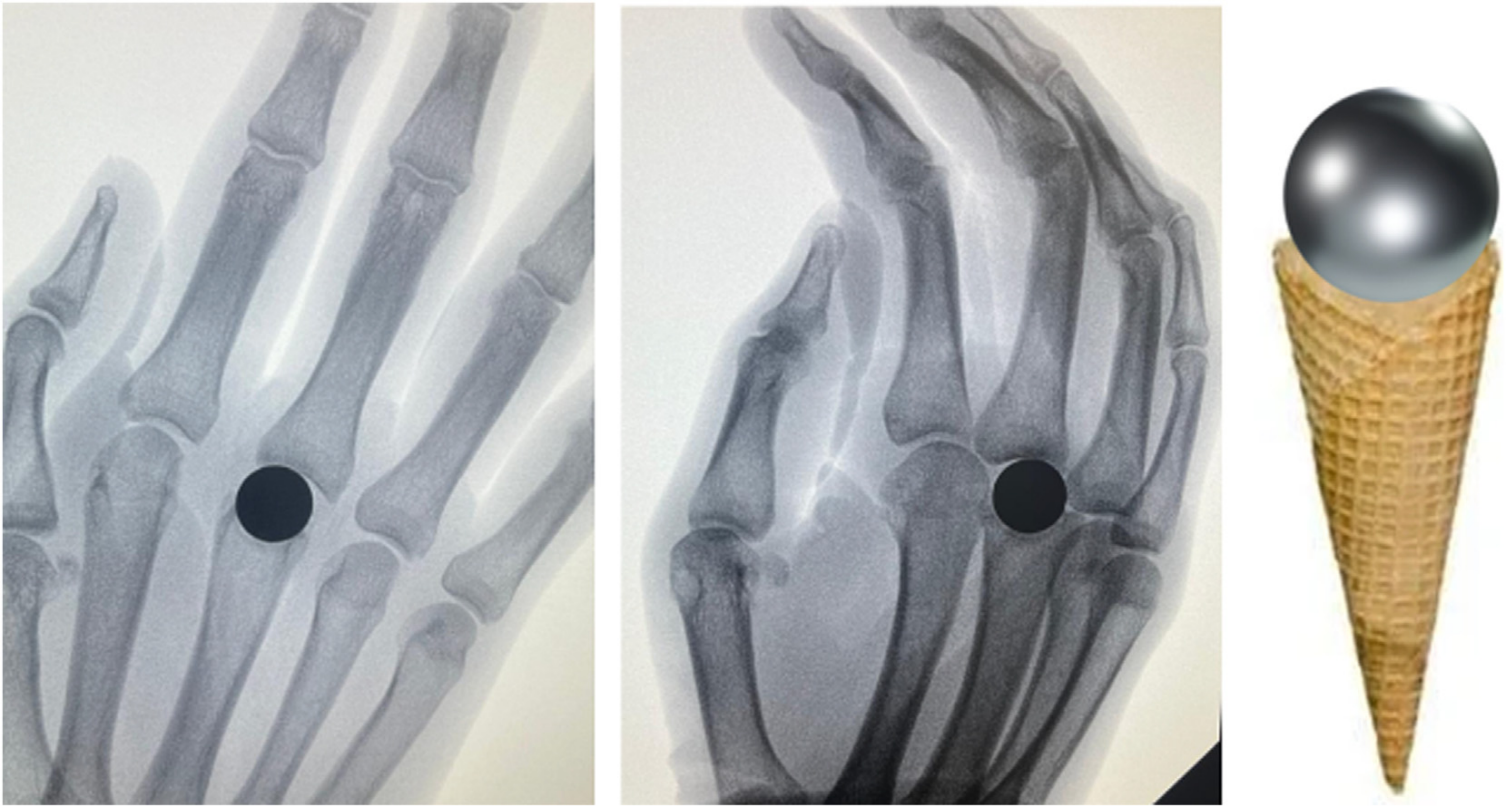
Correct intraoperative positioning of the implant, appearing as a “frozen ball” well-centered within its bony cone and properly aligned.

A trial implant is first inserted (Fig. 1G), followed by intraoperative testing to assess joint stability, which should be self-stable without excessive laxity. Intraoperative fluoroscopic control is systematically performed to verify axial alignment and correct implant positioning, which should appear as a “frozen ball” well-centered within its bony cone and properly aligned (Fig. 2). This step requires careful handling because of the intrinsic fragility of pyrocarbon under torsional and flexion stresses. Excessive force during insertion must be avoided to prevent intraoperative fractures. The final positioning should ensure proper axial centering without tilt or misalignment while guaranteeing optimal primary stability. Intraoperative radiographic control is systematically performed to confirm the absence of fractures or alignment abnormalities. It should be noted that the implant is composed of two materials: pyrocarbon, which is radiolucent, and a graphite core, which is radiopaque and thus clearly visible on fluoroscopy.

The joint capsule is closed with a continuous 3-0 polydioxanone suture (Fig. 1H), ensuring proper implant centering while minimizing the risk of instability. Extensor is sutured using a continuous 3-0 polydioxanone suture over the extensor hood, restoring optimal continuity. The skin is loosely closed with 4-0 sutures to minimize tissue tension and promote optimal healing. A compressive dressing is applied after surgery. Additionally, an intrinsic-plus splint is worn exclusively at night for 3 weeks to allow capsular healing, and syndactyly taping is recommended during daytime for approximately 15 days. Early mobilization under supervised hand therapy begins immediately after surgery.

### Clinical evaluation

Pain was quantified using the VAS.^16^ Range of motion (ROM) in flexion and extension was measured and expressed in degrees. Functional impact was evaluated using the disabilities of the arm, shoulder, and hand score.^17^ Finally, grip strength was measured using a JAMAR dynamometer and expressed in kilograms.

Postoperative complications were also recorded, including dislocation, clicking, instability, infection (sepsis), and complex regional pain syndrome type 1.

### Radiological evaluation

A standard radiographic analysis was performed immediately after surgery and during follow-up at 3, 6, and 12 months, as well as at the final follow-up.

Radiographic assessment was based on several criteria. Implant positioning was analyzed in terms of centering and axial alignment on anteroposterior and lateral views. The implant had to be correctly positioned with strict axial alignment, without excessive tilt (<5° deviation from the longitudinal axis of the metacarpal) or lateral asymmetry. It had to be centered within the bony cone, avoiding excessive cortical contact to prevent inappropriate mechanical stress while maintaining a uniform joint space for optimal articulation kinematics.

Bone remodeling at the metacarpal level was assessed for signs of osteolysis or sclerotic reaction, classified into three categories: no changes, presence of a sclerotic line (radiolucent line), or osteolysis.

Implant integrity was also evaluated to detect potential fractures or structural alterations of the pyrocarbon material.

Proximal migration of the implant was measured on anteroposterior radiographs by comparing the distance between the proximal pole of the implant and the base of the proximal phalanx in the immediate postoperative radiograph. Migration was defined as a progressive decrease in this distance over time, with considerable migration considered when the reduction exceeded 5 mm.^18^

Phalangeal erosion was assessed and classified into four stages (Table 1), adapted from the Sperling classification, as used by Barret et al^19^ to evaluate glenoid erosion in pyrocarbon hemiarthroplasties of the shoulder. Stage 1 corresponds to no visible erosion, stage 2 to erosion limited to the subchondral bone, stage 3 to a hemispherical deformation with progressive distal bone loss, and stage 4 to a major spherical deformation reaching or extending beyond the base of the proximal phalanx.

**Table 1:** Classification of Phalangeal Erosion

Stage	Radiographic Description
Stage 1	No visible erosion
Stage 2	Erosion limited to the subchondral bone
Stage 3	Hemispherical deformation with progressive distal bone loss
Stage 4	Major spherical deformation reaching or extending beyond the base of the proximal phalanx

All radiographs were interpreted by two hand surgery specialists to ensure a consistent and reliable assessment of the data.

### Ethical considerations

This retrospective study was conducted using anonymized data collected from medical records. The study protocol was approved by the Institutional Review Board of the French Society of Orthopaedic Surgery and Traumatology. IRB ethics committee (reference 13-2025). It was conducted in accordance with the principles of the Declaration of Helsinki. The authors adhered to the Strengthening the Reporting of Observational Studies in Epidemiology guidelines (www.strobe-statement.org) in the preparation of this manuscript.

### Statistical analysis

Data were analyzed using the EasyMedStat software application. Comparisons of continuous variables were performed using Student *t* test or the Wilcoxon test, depending on the normality of the distributions. Categorical variables were compared using the chi-square test or Fisher exact tests. A significance threshold of *P* < .05 was considered statistically significant.

## Results

### Demographic data

**Figure 3:**
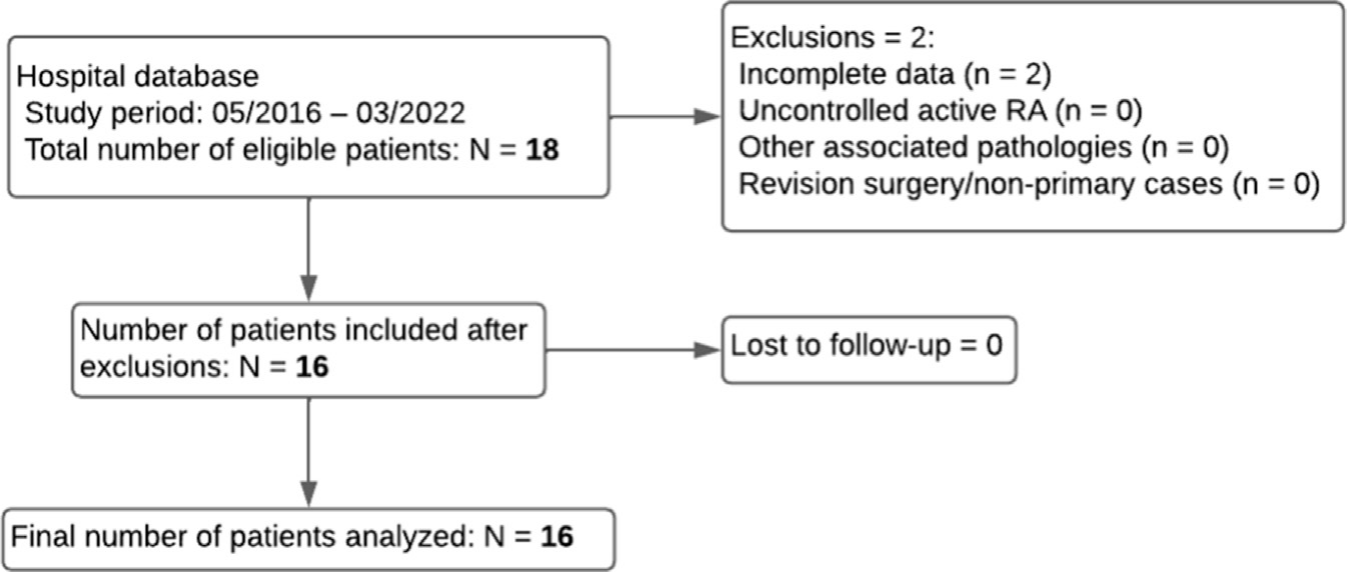
Flowchart of the study.

The Figure 3 presents the flowchart. Our study included 16 patients, with no loss to follow-up. The mean patient age was 60 ± 20 years (Table 2). The mean follow-up duration was 48 ± 21 months. The primary indication for arthroplasty was degenerative osteoarthritis (56.3%).

**Table 2:** Demographic and Clinical Characteristics of the Study Population

Variable	Value
Age (y) mean ± SD (min–max)	60 ± 20 (24–86)
Follow-up (mo) mean ± SD (min–max)	48 ± 21 (24–95)
BMI (kg/m^2^) mean ± SD (min–max)	25.9 ± 5.6 (18–40)
Smoking status n (%)	Yes: 2 (12.5%) | No: 14 (87.5%)
Anticoagulant therapy n (%)	Yes: 2 (12.5%) | No: 14 (87.5%)
Hand dominance n (%)	Right: 12 (85.7%) | Left: 2 (14.3%)
Indications n (%)	Post-traumatic: 4 (25.1%) Degenerative: 11 (68.8%) Postinfectious: 1 (6.3%)
Affected finger	Second: 7 (43.8%) Third: 7 (43.8%) Fourth: 1 (6.3%) Fifth: 1 (6.3%)

min, minimum; max, maximum; BMI, body mass index.

### Functional outcomes

The functional results demonstrated significant improvement, with a reduction in pain (preoperative VAS: 7.4 ± 1 vs postoperative VAS: 0.8 ± 0.9; *P* < .001) and an increase in MCP flexion (preoperative flexion: 41° ± 10° vs postoperative flexion: 64° ± 20°; *P* < .001; Table 3).

**Table 3:** Before surgery and Follow-Up Functional Outcomes

Variable	Before Surgery	Follow-Up	Mean Difference	*P* Value
VAS	7.4 ± 1.0	0.8 ± 0.9	−6.5 ± 1.5	**< .001**
Flexion (°)	41 ± 10	64 ± 20	23 ± 20	**< .001**
Extension loss (°)	22 ± 6	0 ± 6	−18 ± 11	**< .001**
DASH	42.5 ± 11.3	20.2 ± 22.5	19.6 ± 30.2	**.006**
Grip strength (kg)	20.3 ± 7.6	19.0 ± 7.9	1.3 ± 12.5	.68

Bolded *P* values indicate statistical significance (*P* < .05).

DASH, disabilities of the arm, shoulder, and hand.

No major complications were observed in this study. No cases of dislocation, snapping, instability, or postoperative infection were noted.

### Radiological outcomes

**Figure 4:**
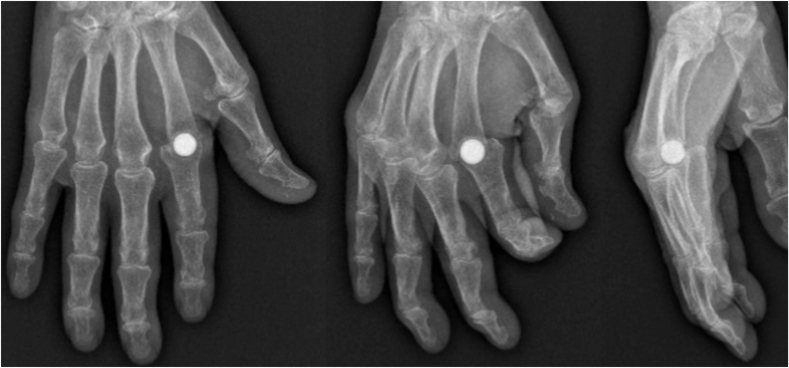
Example of a patient with implant decentering, metacarpal radiolucent line, stage 3 phalangeal erosion, and proximal implant migration.

**Figure 5:**
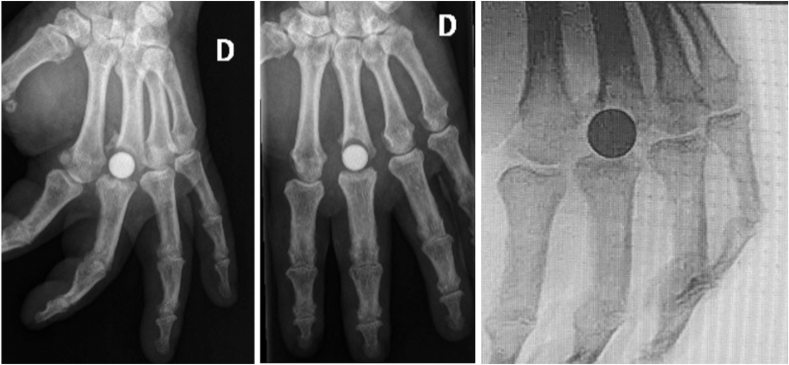
A patient with metacarpal osteolysis at follow-up (left and middle images). The patient was a 77-year-old obese individual (body mass index [BMI] 40). Note the suboptimal implant positioning immediately after surgery (right image).

The radiological results demonstrated a correctly centered implant in 62.5% of the cases immediately after surgery (Table 4; Fig. 4). Regarding metacarpal remodeling, a radiolucent line was observed in 62.5% of the cases (n = 10). One case (6.3%) of metacarpal osteolysis (Fig. 5) was noted without clinical impact.

**Table 4:** Main Radiological Outcomes

Radiological Parameter	Number of Cases (%)
Implant centering	
Yes	10 (62.5%)
No	6 (37.5%)
Metacarpal remodeling	
None	5 (31.2%)
Radiolucent line	10 (62.5%)
Osteolysis	1 (6.3%)
Implant fracture	
Yes	0
No	16 (100%)
Proximal phalanx erosion	
Stage 1	10 (62.5%)
Stage 2	4 (25%)
Stage 3	2 (12.5%)
Stage 4	0
Proximal migration	
Yes	1 (6.2%)
No	15 (93.7%)
Mean ± SD (mm)	0.9 ± 3.5

Regarding phalangeal erosion, most patients (62.5%) showed no signs of erosion (stage 1), whereas 25% (n = 4) presented limited erosion confined to the subchondral bone (stage 2). Two patients (12.5%) demonstrated hemispherical deformation with progressive distal bone loss (stage 3), without notable functional impairment.

Proximal implant migration greater than 5 mm was observed in only one patient (6.2%). This migration was not associated with functional complications or considerable clinical instability.

## Discussion

Osteoarthritis of the MCP joints of the long fingers can lead to considerable pain and functional impairment, sometimes requiring surgical intervention. Pyrocarbon interposition arthroplasty (HAPY implant) represents a therapeutic option aimed at preserving joint mobility while reducing pain. The results of this study demonstrated considerable improvement in MCP ROM, with an increase in flexion from 41° to 64° and a reduction in extension deficit from 22° to 0°, as well as a significant reduction in pain (VAS from 7.4 to 0.8, *P* < .001).^20^, ^21^, ^22^, ^23^

These results are comparable with those reported in published series on Ascension stem pyrocarbon implants, notably Wall and Stern,^14^ observed an increase in the ROM from 62° to 76° and a reduction in mean VAS to 1/10 at the 4-year follow-up.

The study by Daoulas et al,^24^ involving 34 patients treated with the HAPY implant with a mean follow-up of 71.5 months, confirmed pain reduction with VAS scores improving from 7.2 to 1.3, a mean gain in joint flexion of 35°, and an improvement in disabilities of the arm, shoulder and hand scores by 45%. Their study highlighted an 8% complication rate, primarily involving joint snapping and instability requiring specific rehabilitation, but no cases of dislocation or major implant failure (fracture or considerable osteolysis). Our results, although based on a smaller sample size, confirmed satisfactory results of HAPY implant with no complications in one hand surgeon.

Silicone implants provided acceptable functional outcomes but had a high fracture rate (up to 63% in the long-term), although this often does not impact patient satisfaction.^2^^,^^12^ In comparison, pyrocarbon implants offer better mechanical resistance and increased joint stability.^13^^,^^25^

An alternative to implants was interposition arthroplasty using the dorsal capsule or the palmar plate, which provided a similar pain reduction to pyrocarbon implants, although mobility gains were more limited.^5^^,^^26^ However, the absence of an implant decreased the risk of fracture or loosening, making this an interesting option for younger patients or those with low bone density. Pyrocarbon has demonstrated excellent long-term tolerance in various orthopedic indications. The study by Barret et al^27^ reported an 87% survival rate at 10 years for pyrocarbon interposition arthroplasty of the shoulder, with considerable improvement in the Constant score and pain reduction. These findings support our observations regarding MCP arthroplasty, suggesting that pyrocarbon is a reliable and durable material for joint reconstruction, even in joints subjected to high mechanical stress.

No short-term complications were observed in this series, in contrast to some studies reporting implant fractures or migration. Syed et al^28^ described a case of implant fracture 16 months after surgery, attributed to excessive mechanical stress because of abnormal loading following heavy lifting, possibly exacerbated by loosening of the phalangeal implant, and requiring revision to a larger-sized pyrocarbon implant. Similarly, Wagner et al^29^ highlighted an increased intraoperative fracture risk (5.35 times higher with pyrocarbon than with silicone), requiring particular caution during implant insertion. Indeed, pyrocarbon implants must be handled with care, implanted without excessive constraint, and should never meet metallic components (implants, anchors, and metal-ceramic) while ensuring appropriate postoperative follow-up.

The study by Daoulas et al^24^ provided insights into the bone stability of HAPY implants, showing that metacarpal and phalangeal bone erosion measured 0.48 mm and 3.6 mm, respectively, without major clinical consequences. This observation was also reported in other studies with moderate subsidence of pyrocarbon implants, suggesting that prolonged radiographic follow-up is essential to monitor these potential bone changes.^23^

Pyrocarbon had favorable biomechanical and biological properties for MCP arthroplasty, because of its elastic modulus similar to cortical bone, wear resistance, and ability to promote type II collagen expression and cartilage matrix formation with lower bone wear compared with cobalt-chrome.^30^^,^^31^ Its spherical design, without intraosseous fixation, decreased the risk of loosening and the need for revision surgery.

Furthermore, a study by Barret and Boileau^32^ demonstrated that when used as a functional spacer in the treatment of chronic shoulder infections, pyrocarbon did not promote bacterial adhesion. These characteristics may explain the low incidence of infectious and mechanical complications observed in our series.

Radiographic assessment demonstrated implant centering in 62.5% of cases, with a radiolucent line observed in 62.5% of the cases, suggesting progressive bone adaptation to the pyrocarbon implant. Recent experimental studies have shown that this material promotes the adsorption of synovial phospholipids and stimulates chondrogenic cellular activity, leading to the formation of neocartilaginous tissue at the implant interface. Its elastic modulus, closely matching that of cortical bone, allows for a harmonious biomechanical transmission of stresses. It limits bone erosion and supports peri-implant remodeling without causing excessive wear of the surrounding tissues. Proximal migration of more than 5 mm was observed in only one patient (6.2%), with no functional impact. These findings are consistent with those of Wall and Stern,^14^ who reported an average subsidence of 3 mm for pyrocarbon implants, without considerable migration or signs of loosening at the 4-year follow-up. Similarly, Daoulas et al^24^ observed moderate bone subsidence, with an average migration of 0.48 mm at the metacarpal level and 3.6 mm at the phalangeal level, without notable clinical consequences.

Regarding bone tolerance, Simpson-White and Chojnowski^23^ identified periprosthetic erosion in 30% of the cases, which is comparable with the 37.5% of stage 2 and 3 cases observed in our series. Our results did not show any fractures, in contrast to findings reported for silicone implants, where fractures were observed in 42% of the cases at 10 years.^12^

Our study has several strengths. To our knowledge, only one other study has analyzed the outcomes of the HAPY implant.^24^ The fact that all patients were operated on in the same specialized hand surgery center ensures standardized management, thereby reducing bias related to technical variability.

However, our study also has limitations. The sample size remains small (16 patients), which may reduce the statistical power of our results. Although our findings are consistent with other published series, a multicenter study with a larger sample size would help refine these conclusions. Additionally, our mean follow-up of 48 months is insufficient to assess the long-term survival of the implant, particularly regarding the occurrence of fractures, late subsidence, or adjacent degenerative complications.

## Conflicts of Interest

No benefits in any form have been received or will be received related directly to this article.
